# Methyl 2-(1*H*-indole-3-carboxamido)­acetate

**DOI:** 10.1107/S1600536811006660

**Published:** 2011-02-26

**Authors:** Fang Hu, Le Zheng, Xiang Chao Zeng, Kai Ping Li

**Affiliations:** a Department of Chemistry, Jinan University, Guangzhou, Guangdong 510632, People’s Republic of China

## Abstract

The title compound, C_12_H_12_N_2_O_3_, was synthesized by condensation of methyl amino­acetate with 3-trichloro­acetyl­indole. In the crystal, inter­molecular N—H⋯O hydrogen bonds link the mol­ecules into chains parallel to the *b* axis. The chains are further connected into a three-dimensional network by N—H⋯O hydrogen bonds involving the indole N atom. In the molecule, the indole skeleton is nearly planar [maximum deviation = 0.012 (1) Å] and the mean plane of the amido group is twisted from the mean plane of indole ring by 17.2 (1)°.

## Related literature

For the bioactivity of indole derivatives, see: Di Fabio *et al.* (2007 ▶); Sharma & Tepe (2004 ▶). For related structures, see: Huang *et al.* (2009 ▶, 2010 ▶).
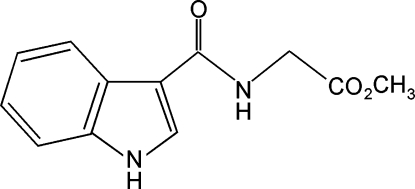


## Experimental

### 

#### Crystal data

C_12_H_12_N_2_O_3_
*M* *_r_* = 232.24
Orthorhombic, 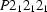

*a* = 8.0024 (2) Å
*b* = 9.1279 (2) Å
*c* = 15.9767 (3) Å
*V* = 1167.02 (4) Å^3^
*Z* = 4
Cu *K*α radiation
μ = 0.80 mm^−1^
*T* = 150 K
0.49 × 0.17 × 0.12 mm

#### Data collection

Oxford Gemini S Ultra area-detector diffractometer
Absorption correction: multi-scan (*CrysAlis PRO*; Oxford Diffraction, 2010) ▶ *T*_min_ = 0.694, *T*_max_ = 0.910
2269 measured reflections
1642 independent reflections
1613 reflections with *I* > 2σ(*I*)
*R*_int_ = 0.016

#### Refinement

*R*[*F* ^2^ > 2σ(*F* ^2^)] = 0.029
*wR*(*F* ^2^) = 0.077
*S* = 1.05
1642 reflections
155 parameters
H-atom parameters constrained
Δρ_max_ = 0.13 e Å^−3^
Δρ_min_ = −0.14 e Å^−3^
Absolute structure: Flack (1983 ▶), 568 Friedel pairs
Flack parameter: −0.2 (3)

### 

Data collection: *CrysAlis PRO* (Oxford Diffraction, 2010 ▶); cell refinement: *CrysAlis PRO*; data reduction: *CrysAlis PRO*; program(s) used to solve structure: *SHELXS97* (Sheldrick, 2008 ▶); program(s) used to refine structure: *SHELXL97* (Sheldrick, 2008 ▶); molecular graphics: *SHELXTL* (Sheldrick, 2008 ▶); software used to prepare material for publication: *SHELXTL*.

## Supplementary Material

Crystal structure: contains datablocks I, global. DOI: 10.1107/S1600536811006660/rz2556sup1.cif

Structure factors: contains datablocks I. DOI: 10.1107/S1600536811006660/rz2556Isup2.hkl

Additional supplementary materials:  crystallographic information; 3D view; checkCIF report

## Figures and Tables

**Table 1:** Hydrogen-bond geometry (Å, °)

*D*—H⋯*A*	*D*—H	H⋯*A*	*D*⋯*A*	*D*—H⋯*A*
N2—H2⋯O1^i^	0.88	2.00	2.8566 (17)	164
N1—H1*A*⋯O2^ii^	0.88	2.15	2.9680 (18)	154

Symmetry codes: (i) 
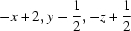
; (ii) 
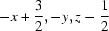
.

**Fig. 1.**
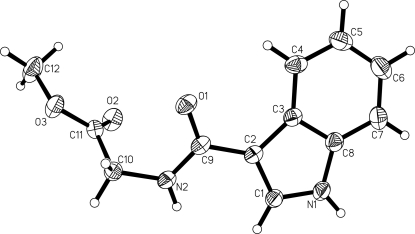
The molecular structure of the title compound, with the atom-numbering scheme. Displacement ellipsoids are drawn at the 30% probability level.

**Fig. 2.**
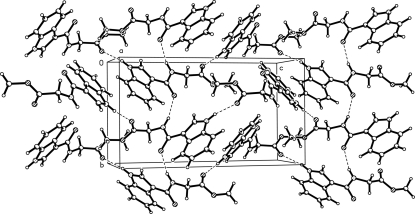
Crystal packing of the title compound viewed approximately along the a axis. Dashed lines indicate hydrogen bonds.

**Fig. 3.**
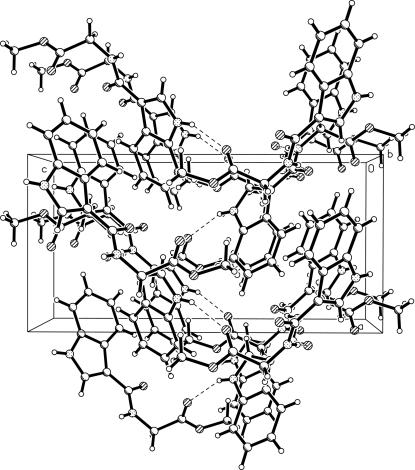
Crystal packing of the title compound viewed along the b axis. Dashed lines indicate hydrogen bonds.

## supplementary crystallographic information

### Comment

Many indole derivatives show important bioactivities, such as metabotropic receptor antagonists (Di Fabio *et al.*, 2007) and protein kinase inhibiting activity (Sharma & Tepe, 2004). This is the reason they have attracted our interest. This study is related to our previous structural investigations of methyl 3-(1-butyl-1*H*-indole-3-carbonyl)aminopropionate (Huang *et al.*, 2009) and methyl 3-(1*H*-indole-3-carbonyl)aminopropionate hemihydrate (Huang *et al.*, 2010).

The molecular structure of the title compound is shown in Fig. 1. In the crystal structure, molecules of the title compound are linked through N2—H2···O1 H-bonds (Table 1) to form chains extending along the *b* axis, which are further connected by N1—HA···O2 H-bonds to form the three-dimensional network (Fig. 2 and Fig. 3). Bond lengths and angles are unexceptional.

### Experimental

The hydrochloric acid salt of methyl aminoacetate (0.63 g, 5 mmol) and 3-trichloroacetylindole (1.32 g, 5 mmol) were added to acetonitrile (10 ml), followed by the dropwise addition of triethylamine (1.2 ml). The mixture was stirred at room temperature for 12 h and then poured into water. After filtration, the precipitate was collected as a yellow solid. The impure product was dissolved in EtOH at room temperature, light yellow orthorhombic crystals suitable for X-ray analysis (m.p. 448 K, 89.2% yield) grew over a period of one week on slow evaporation of the solvent.

### Refinement

All non-H atoms were refined with anisotropic displacement parameters. The H atoms were positioned geometrically [C—H = 0.99Å for CH_2_, 0.98Å for CH_3_, 0.95Å for CH(aromatic) and N—H = 0.88 Å] and refined using a riding model, with *U*_iso_ = 1.2*U*_eq_ (1.5*U*_eq_ for the methyl group) of the parent atom. Friedel pairs were not merged in the refinement

### Crystal data

**Table:**

C_12_H_12_N_2_O_3_	*D*_x_ = 1.322 Mg m^−^^3^
*M**_r_* = 232.24	Melting point: 448 K
Orthorhombic, *P*2_1_2_1_2_1_	Cu *K*α radiation, λ = 1.54178 Å
Hall symbol: P 2ac 2ab	Cell parameters from 1985 reflections
*a* = 8.0024 (2) Å	θ = 4.8–62.6°
*b* = 9.1279 (2) Å	µ = 0.80 mm^−^^1^
*c* = 15.9767 (3) Å	*T* = 150 K
*V* = 1167.02 (4) Å^3^	Prism, light yellow
*Z* = 4	0.49 × 0.17 × 0.12 mm
*F*(000) = 488	

### Data collection

**Table:**

Oxford Gemini S Ultra area-detector diffractometer	1642 independent reflections
Radiation source: fine-focus sealed tube	1613 reflections with *I* > 2σ(*I*)
mirror	*R*_int_ = 0.016
φ and ω scans	θ_max_ = 62.7°, θ_min_ = 5.6°
Absorption correction: multi-scan (*CrysAlis PRO*; Oxford Diffraction, 2010)	*h* = −5→9
*T*_min_ = 0.694, *T*_max_ = 0.910	*k* = −10→8
2269 measured reflections	*l* = −18→15

### Refinement

**Table:**

Refinement on *F*^2^	Secondary atom site location: difference Fourier map
Least-squares matrix: full	Hydrogen site location: inferred from neighbouring sites
*R*[*F*^2^ > 2σ(*F*^2^)] = 0.029	H-atom parameters constrained
*wR*(*F*^2^) = 0.077	*w* = 1/[σ^2^(*F*_o_^2^) + (0.0478*P*)^2^ + 0.0803*P*] where *P* = (*F*_o_^2^ + 2*F*_c_^2^)/3
*S* = 1.05	(Δ/σ)_max_ = 0.011
1642 reflections	Δρ_max_ = 0.13 e Å^−^^3^
155 parameters	Δρ_min_ = −0.14 e Å^−^^3^
0 restraints	Absolute structure: Flack (1983), 568 Friedel pairs
Primary atom site location: structure-invariant direct methods	Flack parameter: −0.2 (3)

### Special details

**Table:**

Geometry. All e.s.d.'s (except the e.s.d. in the dihedral angle between two l.s. planes) are estimated using the full covariance matrix. The cell e.s.d.'s are taken into account individually in the estimation of e.s.d.'s in distances, angles and torsion angles; correlations between e.s.d.'s in cell parameters are only used when they are defined by crystal symmetry. An approximate (isotropic) treatment of cell e.s.d.'s is used for estimating e.s.d.'s involving l.s. planes.
Refinement. Refinement of *F*^2^ against ALL reflections. The weighted *R*-factor *wR* and goodness of fit *S* are based on *F*^2^, conventional *R*-factors *R* are based on *F*, with *F* set to zero for negative *F*^2^. The threshold expression of *F*^2^ > σ(*F*^2^) is used only for calculating *R*-factors(gt) *etc*. and is not relevant to the choice of reflections for refinement. *R*-factors based on *F*^2^ are statistically about twice as large as those based on *F*, and *R*- factors based on ALL data will be even larger.

### Fractional atomic coordinates and isotropic or equivalent isotropic displacement parameters (Å^2^)

**Table:**

	*x*	*y*	*z*	*U*_iso_*/*U*_eq_	
C1	0.8259 (2)	0.08538 (18)	0.12627 (10)	0.0418 (4)	
H1	0.9222	0.0261	0.1184	0.050*	
N1	0.68538 (19)	0.07824 (16)	0.07961 (9)	0.0491 (4)	
H1A	0.6691	0.0180	0.0374	0.059*	
C8	0.5711 (2)	0.17992 (19)	0.10821 (10)	0.0451 (4)	
C3	0.6450 (2)	0.25391 (16)	0.17586 (9)	0.0400 (4)	
C4	0.5524 (2)	0.36258 (18)	0.21720 (11)	0.0498 (4)	
H4	0.5985	0.4145	0.2633	0.060*	
C5	0.3925 (3)	0.3921 (2)	0.18931 (12)	0.0608 (5)	
H5	0.3288	0.4657	0.2167	0.073*	
C6	0.3219 (3)	0.3166 (3)	0.12172 (14)	0.0660 (6)	
H6	0.2115	0.3397	0.1044	0.079*	
C7	0.4094 (3)	0.2099 (2)	0.08007 (12)	0.0593 (5)	
H7	0.3621	0.1586	0.0341	0.071*	
C9	0.9272 (2)	0.22893 (16)	0.25277 (10)	0.0383 (4)	
C10	1.1689 (2)	0.1685 (2)	0.33471 (10)	0.0432 (4)	
H10A	1.2129	0.2687	0.3260	0.052*	
H10B	1.2642	0.0995	0.3315	0.052*	
C11	1.0915 (2)	0.15897 (18)	0.42041 (10)	0.0394 (4)	
C12	1.1129 (3)	0.2444 (3)	0.55958 (11)	0.0767 (7)	
H12A	1.1425	0.1492	0.5839	0.115*	
H12B	0.9915	0.2577	0.5621	0.115*	
H12C	1.1680	0.3226	0.5912	0.115*	
C2	0.8088 (2)	0.19136 (16)	0.18683 (10)	0.0380 (4)	
N2	1.05189 (17)	0.13476 (14)	0.26902 (8)	0.0419 (3)	
H2	1.0620	0.0534	0.2399	0.050*	
O1	0.91340 (16)	0.34378 (12)	0.29446 (7)	0.0476 (3)	
O2	0.97901 (16)	0.07845 (14)	0.43919 (8)	0.0561 (4)	
O3	1.16709 (15)	0.24943 (15)	0.47336 (7)	0.0549 (4)	

### Atomic displacement parameters (Å^2^)

**Table:**

	*U*^11^	*U*^22^	*U*^33^	*U*^12^	*U*^13^	*U*^23^
C1	0.0480 (9)	0.0397 (8)	0.0377 (8)	0.0024 (8)	−0.0025 (8)	−0.0015 (7)
N1	0.0594 (9)	0.0479 (8)	0.0401 (7)	0.0050 (8)	−0.0094 (7)	−0.0091 (7)
C8	0.0508 (10)	0.0442 (9)	0.0404 (9)	0.0019 (8)	−0.0013 (8)	0.0033 (8)
C3	0.0511 (9)	0.0351 (8)	0.0339 (7)	0.0003 (7)	0.0034 (8)	0.0053 (7)
C4	0.0644 (11)	0.0433 (8)	0.0417 (9)	0.0059 (9)	0.0127 (9)	0.0024 (8)
C5	0.0680 (12)	0.0583 (11)	0.0561 (11)	0.0205 (10)	0.0158 (11)	0.0109 (9)
C6	0.0540 (11)	0.0778 (14)	0.0662 (13)	0.0175 (11)	0.0044 (11)	0.0201 (12)
C7	0.0564 (11)	0.0669 (12)	0.0546 (11)	0.0039 (11)	−0.0117 (10)	0.0074 (10)
C9	0.0491 (9)	0.0338 (7)	0.0319 (7)	−0.0079 (7)	0.0056 (7)	0.0023 (7)
C10	0.0386 (8)	0.0511 (9)	0.0398 (8)	−0.0043 (8)	0.0017 (8)	−0.0026 (8)
C11	0.0371 (8)	0.0409 (8)	0.0403 (9)	−0.0010 (8)	−0.0037 (7)	0.0024 (7)
C12	0.0694 (13)	0.1237 (19)	0.0370 (9)	−0.0244 (14)	0.0026 (10)	−0.0143 (12)
C2	0.0483 (9)	0.0319 (8)	0.0337 (8)	−0.0024 (7)	0.0030 (7)	0.0011 (6)
N2	0.0491 (8)	0.0394 (6)	0.0372 (7)	−0.0002 (6)	0.0000 (6)	−0.0061 (6)
O1	0.0636 (7)	0.0346 (5)	0.0446 (6)	−0.0035 (6)	−0.0024 (6)	−0.0080 (5)
O2	0.0574 (7)	0.0629 (7)	0.0481 (7)	−0.0217 (7)	0.0045 (6)	0.0062 (6)
O3	0.0528 (7)	0.0742 (8)	0.0376 (6)	−0.0197 (7)	−0.0002 (6)	−0.0072 (7)

### Geometric parameters (Å, °)

**Table:**

C1—N1	1.351 (2)	C7—H7	0.9500
C1—C2	1.375 (2)	C9—O1	1.2470 (19)
C1—H1	0.9500	C9—N2	1.342 (2)
N1—C8	1.381 (2)	C9—C2	1.458 (2)
N1—H1A	0.8800	C10—N2	1.440 (2)
C8—C7	1.397 (3)	C10—C11	1.505 (2)
C8—C3	1.405 (2)	C10—H10A	0.9900
C3—C4	1.403 (2)	C10—H10B	0.9900
C3—C2	1.440 (2)	C11—O2	1.2002 (19)
C4—C5	1.381 (3)	C11—O3	1.328 (2)
C4—H4	0.9500	C12—O3	1.445 (2)
C5—C6	1.400 (3)	C12—H12A	0.9800
C5—H5	0.9500	C12—H12B	0.9800
C6—C7	1.372 (3)	C12—H12C	0.9800
C6—H6	0.9500	N2—H2	0.8800
			
N1—C1—C2	109.84 (15)	O1—C9—C2	121.73 (15)
N1—C1—H1	125.1	N2—C9—C2	118.18 (13)
C2—C1—H1	125.1	N2—C10—C11	112.53 (13)
C1—N1—C8	109.64 (14)	N2—C10—H10A	109.1
C1—N1—H1A	125.2	C11—C10—H10A	109.1
C8—N1—H1A	125.2	N2—C10—H10B	109.1
N1—C8—C7	129.68 (17)	C11—C10—H10B	109.1
N1—C8—C3	107.41 (15)	H10A—C10—H10B	107.8
C7—C8—C3	122.90 (17)	O2—C11—O3	124.28 (16)
C4—C3—C8	118.67 (16)	O2—C11—C10	124.84 (15)
C4—C3—C2	134.70 (16)	O3—C11—C10	110.86 (14)
C8—C3—C2	106.61 (14)	O3—C12—H12A	109.5
C5—C4—C3	118.38 (18)	O3—C12—H12B	109.5
C5—C4—H4	120.8	H12A—C12—H12B	109.5
C3—C4—H4	120.8	O3—C12—H12C	109.5
C4—C5—C6	121.75 (18)	H12A—C12—H12C	109.5
C4—C5—H5	119.1	H12B—C12—H12C	109.5
C6—C5—H5	119.1	C1—C2—C3	106.50 (14)
C7—C6—C5	121.2 (2)	C1—C2—C9	127.52 (15)
C7—C6—H6	119.4	C3—C2—C9	125.89 (14)
C5—C6—H6	119.4	C9—N2—C10	119.16 (13)
C6—C7—C8	117.10 (19)	C9—N2—H2	120.4
C6—C7—H7	121.4	C10—N2—H2	120.4
C8—C7—H7	121.4	C11—O3—C12	116.79 (14)
O1—C9—N2	120.08 (15)		
			
C2—C1—N1—C8	0.05 (19)	N1—C1—C2—C3	−0.22 (18)
C1—N1—C8—C7	−178.97 (19)	N1—C1—C2—C9	176.50 (15)
C1—N1—C8—C3	0.15 (19)	C4—C3—C2—C1	178.82 (17)
N1—C8—C3—C4	−179.08 (14)	C8—C3—C2—C1	0.30 (17)
C7—C8—C3—C4	0.1 (3)	C4—C3—C2—C9	2.0 (3)
N1—C8—C3—C2	−0.28 (18)	C8—C3—C2—C9	−176.48 (14)
C7—C8—C3—C2	178.92 (16)	O1—C9—C2—C1	166.46 (15)
C8—C3—C4—C5	−0.1 (2)	N2—C9—C2—C1	−14.7 (2)
C2—C3—C4—C5	−178.51 (18)	O1—C9—C2—C3	−17.4 (2)
C3—C4—C5—C6	0.2 (3)	N2—C9—C2—C3	161.42 (15)
C4—C5—C6—C7	−0.2 (3)	O1—C9—N2—C10	−0.2 (2)
C5—C6—C7—C8	0.2 (3)	C2—C9—N2—C10	−179.07 (14)
N1—C8—C7—C6	178.87 (18)	C11—C10—N2—C9	69.72 (19)
C3—C8—C7—C6	−0.1 (3)	O2—C11—O3—C12	2.6 (3)
N2—C10—C11—O2	30.2 (2)	C10—C11—O3—C12	−175.89 (17)
N2—C10—C11—O3	−151.31 (14)		

### Hydrogen-bond geometry (Å, °)

**Table:**

*D*—H···*A*	*D*—H	H···*A*	*D*···*A*	*D*—H···*A*
N2—H2···O1^i^	0.88	2.00	2.8566 (17)	164
N1—H1A···O2^ii^	0.88	2.15	2.9680 (18)	154

Symmetry codes: (i) −*x*+2, *y*−1/2, −*z*+1/2; (ii) −*x*+3/2, −*y*, *z*−1/2.
